# Autonomic effects and ablation success after pulsed field and cryoballoon ablation: a SINGLE SHOT CHAMPION substudy

**DOI:** 10.1093/europace/euag152

**Published:** 2026-06-18

**Authors:** Laurent Roten, Thomas Kueffer, Patrick Badertscher, Peter Jüni, Sven Knecht, Gregor Thalmann, Nikola Kozhuharov, Philipp Krisai, Salik Ur Rehman Iqbal, Corinne Jufer, Jens Maurhofer, Dik Heg, Tiago V Pereira, Felix Mahfoud, Helge Servatius, Hildegard Tanner, Michael Kühne, Christian Sticherling, Tobias Reichlin

**Affiliations:** Department of Cardiology, Inselspital, Bern University Hospital, University of Bern, Freiburgstrasse, Bern CH-3010, Switzerland; Department of Cardiology, Inselspital, Bern University Hospital, University of Bern, Freiburgstrasse, Bern CH-3010, Switzerland; Department of Cardiology, University Hospital Basel, University of Basel, Basel, Switzerland; Clinical Trial Service Unit and Epidemiological Studies Unit, Nuffield Department of Population Health, University of Oxford, Oxford, UK; Department of Cardiology, University Hospital Basel, University of Basel, Basel, Switzerland; Department of Cardiology, Inselspital, Bern University Hospital, University of Bern, Freiburgstrasse, Bern CH-3010, Switzerland; Department of Cardiology, Inselspital, Bern University Hospital, University of Bern, Freiburgstrasse, Bern CH-3010, Switzerland; Department of Cardiology, University Hospital Basel, University of Basel, Basel, Switzerland; Department of Cardiology, Inselspital, Bern University Hospital, University of Bern, Freiburgstrasse, Bern CH-3010, Switzerland; Department of Cardiology, Inselspital, Bern University Hospital, University of Bern, Freiburgstrasse, Bern CH-3010, Switzerland; Department of Cardiology, Inselspital, Bern University Hospital, University of Bern, Freiburgstrasse, Bern CH-3010, Switzerland; Department of Clinical Research, University of Bern, Bern, Switzerland; Clinical Trial Service Unit and Epidemiological Studies Unit, Nuffield Department of Population Health, University of Oxford, Oxford, UK; Department of Cardiology, University Hospital Basel, University of Basel, Basel, Switzerland; Department of Cardiology, Inselspital, Bern University Hospital, University of Bern, Freiburgstrasse, Bern CH-3010, Switzerland; Department of Cardiology, Inselspital, Bern University Hospital, University of Bern, Freiburgstrasse, Bern CH-3010, Switzerland; Department of Cardiology, University Hospital Basel, University of Basel, Basel, Switzerland; Department of Cardiology, University Hospital Basel, University of Basel, Basel, Switzerland; Department of Cardiology, Inselspital, Bern University Hospital, University of Bern, Freiburgstrasse, Bern CH-3010, Switzerland

**Keywords:** Autonomic nervous system, Ganglionated plexi, Pulsed field ablation, Cryoballoon ablation, Heart rate variability, Pulmonary vein isolation, Paroxysmal atrial fibrillation, Randomized controlled trial

## Abstract

**Aims:**

Pulmonary vein isolation (PVI) using radiofrequency and cryoballoon ablation (CBA) modulates the cardiac autonomic nervous system (ANS) by affecting epicardial ganglionated plexi. In contrast, pulsed field ablation (PFA) appears to exert minimal autonomic effects. The impact of cardiac ANS modulation on arrhythmia recurrence remains incompletely understood.

**Methods and results:**

In this multicentre trial substudy, 210 patients with symptomatic paroxysmal atrial fibrillation (AF) were randomized 1:1 to PVI using PFA or CBA. All patients received an implantable cardiac monitor at the time of ablation. Daytime heart rate (DHR), night-time heart rate (NHR), and heart rate variability (HRV) were continuously recorded over 12 months and analysed at Days 1–2, Months 3, 6, 9, and 12 post-ablation, and related to arrhythmia-free outcome. PFA (*n* = 105) was associated with higher HRV at Days 1–2 compared to CBA [*n* = 105; 100 (79–122) ms vs. 73 (59–93) ms, *P* < 0.001]. This trend persisted throughout follow-up. DHR and NHR were also consistently lower in the PFA group. Freedom from arrhythmia recurrence at 12 months was higher with PFA than CBA (63% vs. 49%, *P* = 0.046). HRV did not differ between PFA patients with and without recurrence. A stronger initial HRV suppression predicted favourable outcomes in CBA patients (67 ms vs. 84 ms in patients without vs. with arrhythmia recurrence, *P* = 0.002).

**Conclusion:**

Despite reduced cardiac ANS modulation, PFA was associated with a superior arrhythmia-free outcome compared to CBA. These findings suggest that cardiac ANS modulation may not be critical for the successful ablation of paroxysmal AF.

## Graphical Abstract

**Graphical Abstract:**
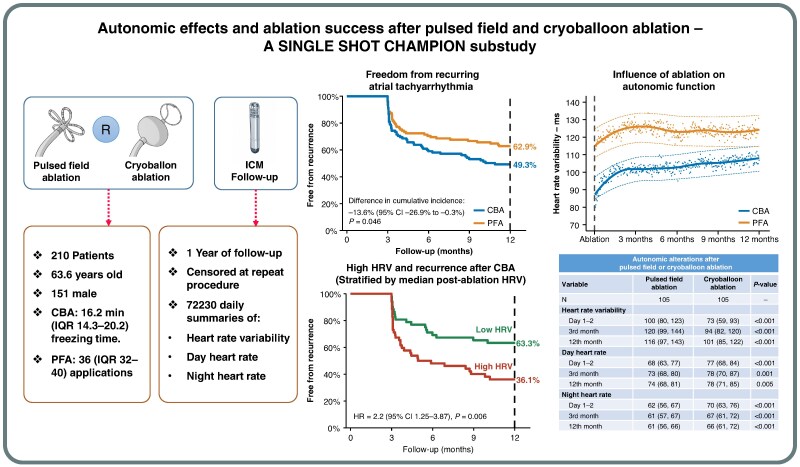

## Introduction

Pulmonary vein isolation (PVI) using thermal energy sources such as radiofrequency or cryoballoon ablation (CBA) is an established treatment for symptomatic paroxysmal atrial fibrillation (AF), demonstrating superiority over antiarrhythmic drug therapy. International guidelines recommend catheter ablation as a first-line therapy (Class I, Level A) for rhythm control in these patients.^1,2^

Beyond isolating the pulmonary veins (PVs), thermal ablation also affects the cardiac autonomic nervous system (ANS), by incidentally ablating ganglionated plexi (GP) at the atrial–PV junction.^3,4^ Whether such autonomic modulation contributes to long-term ablation success remains debated.^5,6^ Some studies, including the CIRCA-DOSE trial and related analyses, demonstrated that thermal ablation induces sustained changes in heart rate variability and nocturnal heart rate, which correlated with freedom from arrhythmia recurrence.^7,8^ In contrast, the AFACT trial found no benefit from surgical GP ablation and reported more adverse events, including sinus node dysfunction and pacemaker implantation.^9^

Pulsed field ablation (PFA), a newer non-thermal ablation modality, offers tissue selectivity, ablating myocardium while sparing neural and vascular structures.^10^ In the SINGLE SHOT CHAMPION trial, PFA was noninferior—and statistically superior—to CBA for arrhythmia recurrence, as assessed through continuous rhythm monitoring.^11^

Given its tissue selectivity, PFA may induce less ANS modulation than thermal ablation. This raises questions about the role of ANS modulation for effective AF ablation. In this study, we examined autonomic changes after PFA or CBA in the SINGLE SHOT CHAMPION trial and evaluate whether ANS modulation is associated with arrhythmia-free outcomes in patients with paroxysmal AF.

## Methods

### Trial design

This is a sub-study of SINGLE SHOT CHAMPION (NCT05534581)—a multicentre, prospective, parallel-group, single-blind randomized clinical trial with blinded endpoint assessment, conducted at two clinical centres in Switzerland. Details of the study protocol have been outlined previously.^12^ The trial design and execution were overseen by an academic steering committee. Data monitoring, collection, and primary analyses were conducted under the supervision of the Clinical Trials Unit Bern, with support from the steering committee. The authors affirm the accuracy of the data presented. Data supporting the findings of this analysis are available from the corresponding author upon reasonable request.

### Study participants and randomization

Eligible participants were adults with symptomatic paroxysmal AF. Patients were randomized in a 1:1 ratio to undergo PVI using either PFA or CBA. Randomization was performed using a centralized, computer-generated system, and patients were stratified by trial centre.

### Ablation procedure

PVI was performed according to standard clinical practice. Sedation was performed using propofol, fentanyl, and midazolam while high-risk patients underwent general anaesthesia. For patients randomized to PFA, ablation was performed using a multielectrode pentaspline PFA catheter (FARAPULSE, Boston Scientific, Marlborough, MA, USA). Eight PFA applications using 2 kV pulses were delivered as the standard lesion set for each PV, comprising two pairs of two applications in basket- and flower-like configurations, with a 30–40°catheter rotation between ablation pairs. Additional PFA applications were delivered based on electrograms to achieve PVI. PVI was verified at the end of the procedure using the PFA catheter in basket configuration in all PVs to assess entrance and exit block using 10 V × 2 ms pacing across all adjacent-electrode pairs. For those randomized to CBA, PVI was achieved using a cryoballoon catheter (Arctic Front Advance, Medtronic, Medtronic, MN, USA). In case of an effective freeze (defined as disappearance of all local PV signals or reaching −40 °C within 60 s in the absence of local signals), cryoablation was continued for two additional minutes. If the freeze was ineffective (no PVI or failure to reach the target temperature in the absence of local signals), the balloon and/or guidewire were repositioned to improve catheter orientation, and a new lesion was applied to achieve PVI. PVI was verified at the end of the procedure using the circular mapping catheter (Achieve, Medtronic, MN, USA) in all PVs to assess entrance and exit block using 10 V × 2 ms pacing across all adjacent-electrode pairs. Additional left atrial lesions were not allowed in either group. Antiarrhythmic drugs were permitted during the 3-month blanking period, but discontinued thereafter.

### Rhythm monitoring

All participants received an implantable cardiac monitor (ICM; Reveal LinQ, Medtronic, MN, USA) at the end of the ablation procedure. ICMs were programmed with standardized settings to monitor arrhythmias and autonomic markers, including heart rate variability (HRV), daytime heart rate (DHR), and night-time heart rate (NHR). HRV was quantified by the ICM as the standard deviation of mean inter-beat intervals during 5-min segments across 24 h, analogous to standard deviation of the average NN (or RR) intervals (SDANN).^13^ DHR and NHR are defined by the ICM as the average heart rates during daytime (8:00 a.m.—8:00 p.m.) and night-time (00:00 a.m. – 4:00 a.m.), respectively. Calculations excluded periods of active AF, during which mean and maximum heart rates and AF burden (hours per day) were recorded. The ICM reports a single DHR, NHR, and HRV value per day, precluding frequency-domain analysis.

### Outcome events

The primary outcome of interest was the comparison of autonomic effects between PFA and CBA groups, assessed by magnitude of daily HRV, DHR, and NHR. Median values of autonomic parameters within groups were analysed at Days 1–2, Month 3 (Days 61–91), and Month 12 (Days 335–365) post-ablation. In cases of repeat ablation, patients were censored on the day of the procedure. Secondary outcomes included AF recurrence—defined as any documented atrial tachyarrhythmia (AF, atrial flutter, or atrial tachycardia) lasting >30 s beyond a blanking period of 90 days—and their correlation with HRV, DHR, and NHR. Further, AF recurrence was analysed by stratifying the PFA and CBA groups into high and low post-ablation HRV categories, using the group-specific median as the cut-off.

### Statistical analysis

Baseline data are represented as mean ± standard deviation or median [interquartile range (IQR)), depending on the variable’s distribution. Autonomic data is presented as median (IQR). Statistical analyses included Fisher’s exact test for categorical variables and the Wilcoxon rank-sum and the Mann–Whitney *U* test for continuous variables. Trends over time were explored using locally estimated scatterplot smoothing (LOESS). Arrhythmia-free survival was estimated using the Kaplan–Meier method. A Cox proportional hazards model was used to estimate the effect of HRV on arrhythmia recurrence. All statistical tests were two-sided and were considered statistically significant at *P* < 0.05. Statistical analyses were performed using R 4.5.0 (R Core Team, Vienna, Austria).

## Results

### Patients and interventions

From September 2022 to November 2023, 210 patients were randomly assigned to undergo PFA (105 patients) or CBA (105 patients). The mean patient age was 64 years, and 28% were women. Baseline characteristics were balanced between the groups (*Table 1*). For PVI, the median number of applications in the PFA group was 36 (IQR 32–40), while the CBA group had a median of 5 freezes (IQR 5–6) and a mean total freezing time of 16.2 (IQR 14.3–20.2) min. The 31 mm catheter was used in 104 (99%) PFA procedures (a 35 mm catheter in one case), and a 28 mm balloon was used in 104 (99%) CBA procedures (a 23 mm balloon in one case). Cavo-tricuspid isthmus RF ablation was performed in 14 patients (13.3%) in the PFA group and 12 patients (11.4%) in the CBA group. General anaesthesia was used in 5 (4.8%) patients in the PFA group and 7 (6.7%) patients in the CBA group.

**Table 1:** Characteristics of the patients at baseline^a^

	Overall	Pulsed field ablation	Cryoballoon ablation
*n*	210	105	105
Age—years	63.6 (9.5)	64.0 (9.4)	63.3 (9.6)
Sex, male	151 (71.9)	77 (73.3)	74 (70.5)
Body-mass index—kg/m^2^	27.2 (4.3)	27.0 (3.9)	27.3 (4.7)
Current tobacco use	22 (10.5)	10 (9.5)	12 (11.4)
CHA2DS2-VA score [IQR]^b^	1.0 [1.0, 2.0]	1.0 [1.0, 2.0]	1.0 [1.0, 2.0]
Concomitant clinical conditions			
Hypertension	114 (54.3)	56 (53.3)	58 (55.2)
Vascular disease	28 (13.3)	12 (11.4)	16 (15.2)
History of congestive heart failure	12 (5.7)	9 (8.6)	3 (2.9)
Diabetes	22 (10.5)	13 (12.4)	9 (8.6)
Previous stroke, TIA, or peripheral embolism	11 (5.2)	4 (3.8)	7 (6.7)
Obstructive sleep apnoea syndrome	28 (13.3)	14 (13.3)	14 (13.3)
Antiarrhythmic drugs^c^	46 (21.9)	22 (21.0)	24 (22.9)
Echocardiographic data			
Left atrial volume index—mL/m^2d^	34.9 (10.5)	34.5 (9.6)	35.4 (11.3)
Left ventricular ejection fraction—%	59.6 (6.0)	59.7 (6.5)	59.5 (5.5)
Systolic blood pressure—mmHg	133.5 (16.8)	134.9 (16.2)	132.0 (17.4)
Diastolic blood pressure—mmHg	75.9 (11.7)	75.9 (12.9)	75.9 (10.4)
RR interval on pre-ablation electrogram^e^	58.0 (12.3)	59.7 (12.5)	56.3 (11.9)

^a^Values are No. (%) or mean (SD) as appropriate. IQR, interquartile range; TIA, transient ischaemic attack.

^b^The CHA2DS2-VA score is an assessment of the risk of stroke among patients with atrial fibrillation and ranges from 0 to 8, with higher scores indicating a higher risk of stroke.

^c^A complete list of antiarrhythmic and other drugs can be found in the Supplementary material online, *Table S1*.

^d^Left atrial volume index data were available for 98 Pulsed field ablation and 96 Cryoballoon ablation patients.

^e^Recorded on the day of or the day before the ablation procedure.

Two patients in the CBA group had premature explanation of their ICM at 214 and 239 days, in one case due to patient preference, the other due to medical reasons. Data were censored after removal. Twenty-six patients (10 from the CBA group and 16 from the PFA group) underwent a repeat procedure due to arrhythmia recurrence during the follow-up period. They were censored at the date of their repeat procedure. One patient from the PFA group was managed abroad, prohibiting daily ICM readouts.

### Autonomic markers

**Figure 1:**
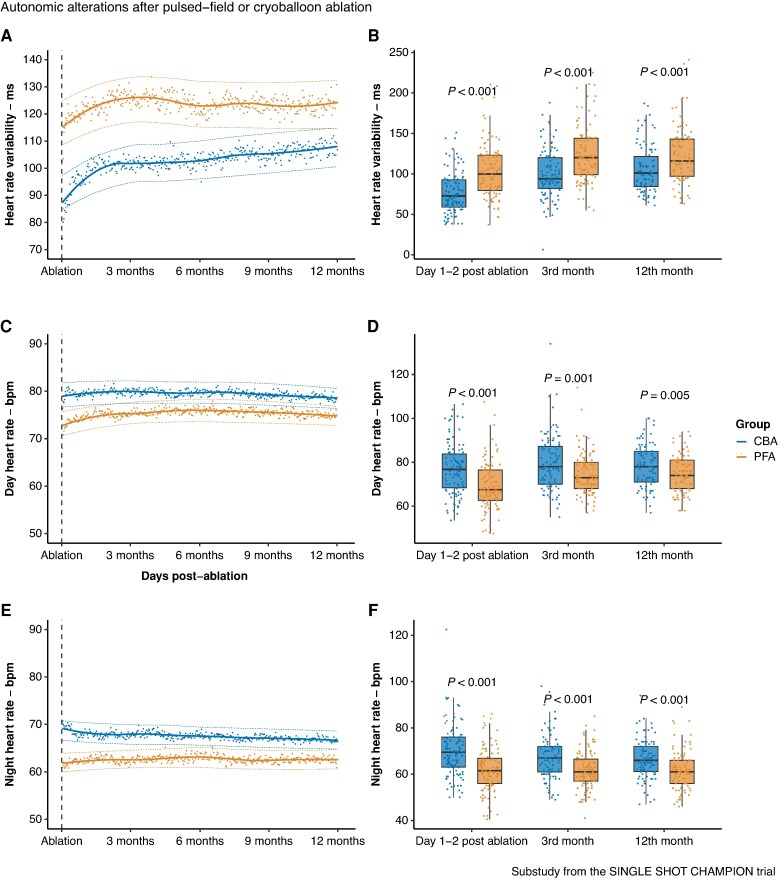
Autonomic alterations after pulsed field or cryoablation. Panels A, C, and E show scatter plots of daily mean (mean of *n* = 105 per group per day) heart rate variability, daytime heart rate, and night-time heart rate over 12 months, with LOESS smoothing and 95% confidence interval bands. Panels B, D, and F show median values per patient within each follow-up window (*n* = 105 per group). Pulsed field ablation consistently resulted in less autonomic modulation across all markers and follow-up windows. CBA, cryoballoon ablation; PFA, pulsed field ablation.

From the ICMs, 72230 daily summaries were available for analysis (mean of 344 summaries per patient). On Days 1–2 post-ablation, median HRV was higher in PFA patients compared to CBA patients [100 (IQR 80–123) ms vs. 73 (IQR 59–93) ms, *P* < 0.001]. This finding was consistent across follow-up windows at Month 3 [120 (IQR 99–144) ms for PFA vs. 94 (IQR 82–129) ms for CBA, *P* < 0.001] and Month 12 [116 (97–143) ms for PFA vs. 101 (85–122) ms for CBA, *P* < 0.001, *Figure 1* and *Table 2*). Quantitative temporal analysis can be found in Supplementary material online, *Table S2*. are Corresponding to the effect on HRV, DHR and NHR were consistently lower in the PFA group through follow-up.

**Table 2:** Autonomic alterations after pulsed field and cryoballoon ablation^a^

Variable	Pulsed field ablation	Cryoballoon ablation	*P*
*n*	105	105	
**Heart rate variability**			
Day 1–2^b^	100 [80, 123]	73 [59, 93]	<0.001
Third month^c^	120 [99, 144]	94 [82, 120]	<0.001
6th month^d^	118 [99, 143]	95 [81, 124]	<0.001
9th month^e^	117 [104, 138]	100 [83, 120]	<0.001
12th month^f^	116 [97, 143]	101 [85, 122]	<0.001
**Day heart rate**			
Days 1–2^b^	68 [63, 77]	77 [68, 84]	<0.001
3rd month^c^	73 [68, 80]	78 [70, 87]	0.001
6th month^d^	74 [68, 82]	78 [72, 86]	0.013
9th month^e^	74 [68, 81]	77 [71, 84]	0.016
12th month^f^	74 [68, 81]	78 [71, 85]	0.005
**Night heart rate**			
Days 1–2^b^	62 [56, 67]	70 [63, 76]	<0.001
3rd month^c^	61 [57, 67]	67 [61, 72]	<0.001
6th month^d^	61 [57, 67]	66 [60, 72]	<0.001
9th month^e^	61 [57, 66]	66 [59, 71]	<0.001
12th month^f^	61 [56, 66]	66 [61, 72]	<0.001

^a^Numbers are milliseconds [IQR] for heart rate variability and beats per minute [IQR] for day heart rate and night heart rate. IQR denotes interquartile range. *P*-values indicate differences between the cryoballoon and pulsed field ablation groups.

^b^Median of Day 1 and Day 2 post-ablation

^c^Median of Day 61 and Day 91 post-ablation

^d^Median of Day 153 and Day 183 post-ablation

^e^Median of Day 244 and Day 274 post-ablation

^f^Median of Day 335 and Day 365 post-ablation

### Arrhythmia recurrence

**Figure 2:**
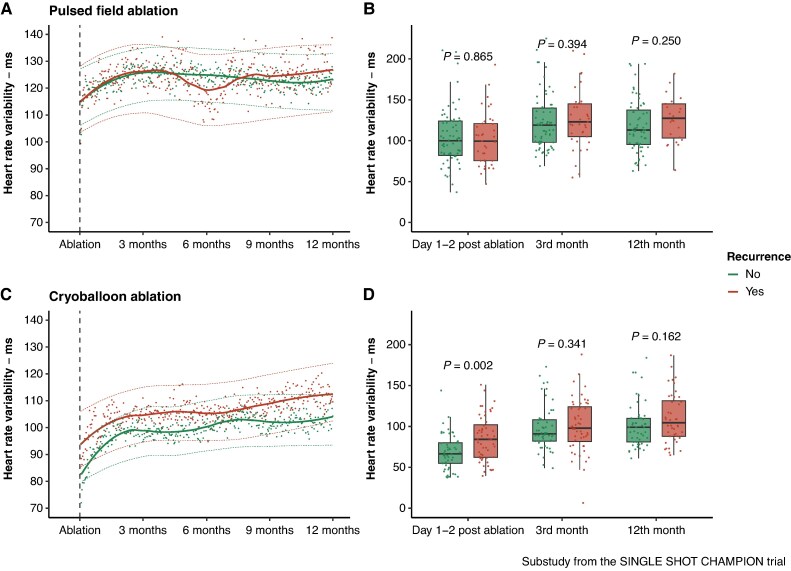
Autonomic alterations in patients with and without recurrence. Autonomic alterations in patients with and without arrhythmia recurrence after pulmonary vein isolation. Panels A and C show scatter plots of daily mean values (mean of *n* = 105 per group per day) with LOESS smoothing and 95% confidence intervals; Panels B and D display median values per patient within each follow-up window (*n* = 105 per group).

Previously reported freedom from recurrent atrial tachyarrhythmia at 1 year using a blanking period of 90 days was 63% with PFA and 49% with CBA (absolute difference −13.6; 95% confidence interval, −26.9 to −0.3; *P* < 0.001 for noninferiority, *P* = 0.046 for superiority, Supplementary material online, *Figure S1*).^11^ No differences in autonomic function markers were observed in patients with and without recurrences in the PFA group. In contrast, patients in the CBA group with subsequent recurrence showed higher HRV 1–2 days post-ablation (67 ms vs. 84 ms, *P* = 0.002, *Figure 2*). This HRV difference diminished during later follow-up periods. DHR and NHR remained similar in the PFA group regardless of recurrence, whereas patients with recurrence in the CBA group exhibited reduced DHR at Months 6 and 9 after ablation (*Table 3*).

**Table 3:** Autonomic data in patients with and without recurrence^a^

Variable	No recurrence	Recurrence	*P*
**PFA—Heart rate variability**			
Days 1–2^b^	100 [82, 124]	100 [76, 121]	0.865
3rd month^c^	119 [98, 141]	123 [105, 145]	0.394
6th month^d^	119 [101, 144]	118 [94, 143]	0.633
9th month^e^	116 [99, 133]	120 [108, 142]	0.515
12th month^f^	113 [96, 141]	128 [103, 145]	0.250
**PFA—Day heart rate**			
Days 1–2^b^	68 [63, 75]	69 [64, 79]	0.450
3rd month^c^	72 [67, 81]	73 [70, 78]	0.765
6th month^d^	75 [68, 82]	72 [69, 82]	0.759
9th month^e^	75 [68, 82]	72 [67, 79]	0.254
12th month^f^	76 [68, 81]	73 [66, 78]	0.166
**PFA—Night heart rate**			
Days 1–2	61 [56, 66]	63 [57, 69]	0.405
3rd month^b^	62 [57, 67]	60 [59, 66]	0.942
6th month^c^	62 [57, 67]	61 [58, 65]	0.741
9th month^d^	62 [56, 67]	60 [57, 65]	0.482
12th month^e^	62 [57, 67]	60 [54, 64]	0.147
**CBA—Heart rate variability**			
Days 1–2^b^	67 [55, 80]	84 [62, 102]	0.002
3rd month^c^	91 [82, 108]	98 [82, 124]	0.341
6th month^d^	94 [81, 104]	98 [82, 129]	0.274
9th month^e^	98 [80, 111]	104 [84, 133]	0.299
12th month^f^	99 [81, 110]	105 [88, 132]	0.162
**CBA—Day heart rate**			
Days 1–2^b^	79 [70, 85]	74 [67, 81]	0.119
3rd month^c^	79 [74, 88]	77 [69, 86]	0.172
6th month^d^	80 [75, 87]	78 [70, 82]	0.038
9th month^e^	80 [72, 87]	75 [69, 82]	0.028
12th month^f^	80 [72, 87]	77 [70, 83]	0.079
**CBA—Night heart rate**			
Days 1–2^b^	71 [66, 77]	67 [60, 74]	0.048
3rd month^c^	67 [63, 74]	65 [60, 71]	0.145
6th month^d^	67 [64, 74]	66 [59, 71]	0.086
9th month^e^	67 [62, 75]	65 [58, 70]	0.110
12th month^f^	67 [62, 74]	65 [60, 70]	0.078

^a^Numbers are milliseconds for Heart rate variability and beats per minute for day heart rate and night heart rate. CBA denotes cryoballoon ablation and PFA pulsed field ablation. *P*-values indicate differences between the cryoballoon and pulsed field ablation groups.

^b^Median of Day 1 and Day 2 post-ablation

^c^Median of Day 61 and Day 91 post-ablation

^d^Median of Day 153 and Day 183 post-ablation

^e^Median of Day 244 and Day 274 post-ablation

^f^Median of Day 335 and Day 365 post-ablation

**Figure 3:**
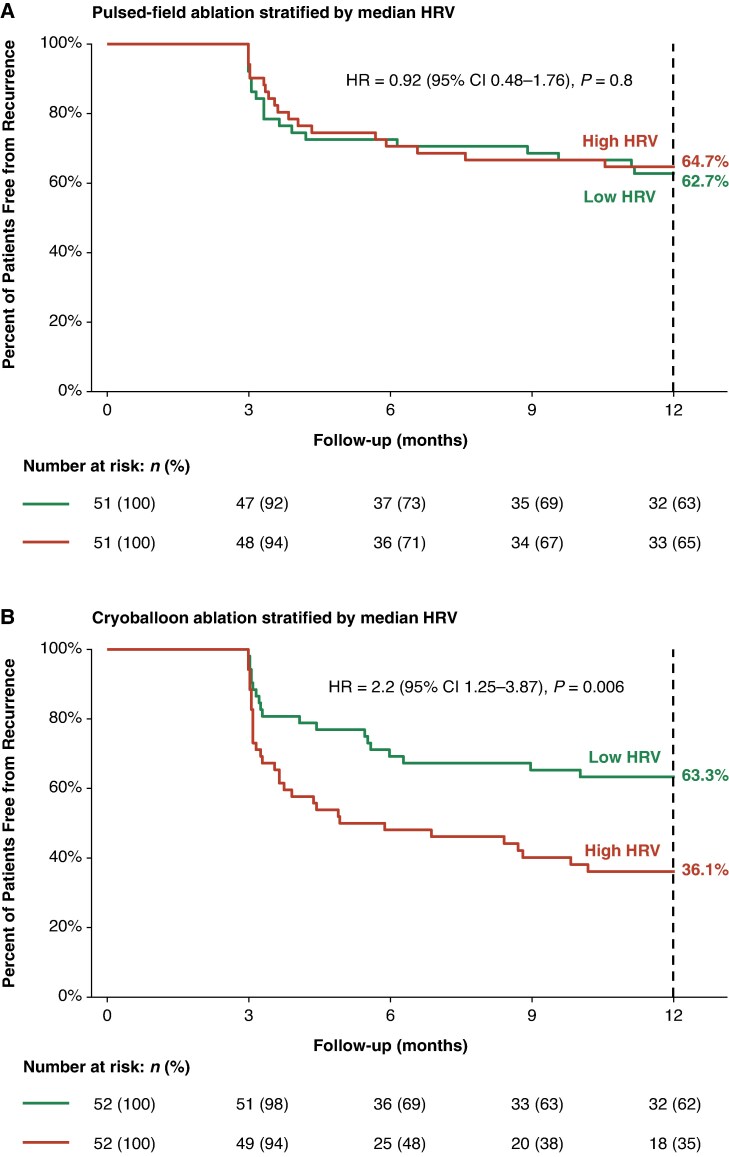
Heart rate variability and recurrence-free survival. Recurrence-free survival in pulsed-field (panel A) and cryoballoon ablation (panel B) patients stratified by median heart rate variability (HRV) on Days 1–2 after ablation. Low HRV: HRV < median HRV; High HRV: HRV > median.

Among patients treated with CBA, lower HRV (i.e. HRV below the group-specific median) on Days 1–2 post-procedure was associated with significantly fewer arrhythmia recurrences during follow-up (37% vs. 64%, HR 2.20, 95%CI 1.25–3.87, *P* = 0.006, *Figure 3*). No such difference was observed among patients treated with pulsed-field ablation. CBA ablation characteristics were not different between the high- and low- HRV groups (see Supplementary material online, *Table S3*).

## Discussion

Continuous rhythm monitoring after PVI in patients with paroxysmal AF revealed distinct differences in cardiac ANS modulation between PFA and CBA.

1. PFA was associated with significantly reduced cardiac ANS modulation compared to CBA
2. Despite reduced cardiac ANS modulation, PFA was superior to CBA regarding arrhythmia-free survival.
3. Among CBA-treated patients, higher early HRV was associated with an increased risk of arrhythmia recurrence.
4. In contrast, cardiac ANS modulation after PFA did not differ between patients with and without recurrence.

Our results in the CBA group closely resemble those reported by Tang *et al*. in the CIRCA-DOSE study, using the same ICM for evaluation of autonomic function post-ablation.^8^ Specifically, HRV values [61 ms (IQR48–78)], as well as DHR [73 bpm (IQR66–80)], and NHR [69 bpm (IQR 63–78)] after ablation are comparable with our cohort. This suggests a similar degree of cardiac ANS modulation following CBA and confirms that our patients received treatment consistent with contemporary CBA protocols.

In contrast, patients treated with PFA exhibited consistently lower DHR and NHR and significantly higher HRV values throughout follow-up. A substudy of the ADVENT trial similarly found modest, transient ANS modulation following PFA, compared to more pronounced effects observed after thermal ablation.^14^ These findings indicate reduced cardiac ANS modulation, consistent with a weaker impact of PFA on the GP.

HRV should be interpreted cautiously as a surrogate of sympatho–vagal balance, as time-domain measures such as SDANN are influenced by mean heart rate through cycle-length dependence. Because the ILR stores only a single daily HRV summary value without individual R–R intervals or short-term segments, frequency-domain analyses, and meaningful heart rate–adjusted correction were not feasible; HRV was therefore interpreted alongside daytime and night-time heart rate measures.

The weaker impact of PFA on the GP is in line with the known tissue selectivity of PFA, which spares neuronal structures compared to thermal ablation technologies such as CBA.^4,15,16^ Indeed, a 2025 pooled analysis finds lower heart rate increase after PFA compared to thermal ablation, a finding that was consistent mid- and long-term (12 months) and across age, set, and different thermal ablation modalities.^17^ Experimental and biomarker studies further support the observation that PFA exerts reduced impact on the cardiac ANS. In a study comparing PFA using a lattice-tip catheter with radiofrequency ablation, extracardiac vagal stimulation revealed significantly less suppression of sinoatrial and atrioventricular node responses after PFA, particularly during ablation near the right PVs.^18^ Autonomic function also recovered more rapidly following PFA, indicating a weaker and more transient effect of PFA on autonomic function. In a study measuring the neuronal injury biomarker S100B, serum levels of S100B increased after PVI in both PFA and CBA groups, but were fourfold higher following CBA, even after excluding patients with cerebral emboli.^19^

Despite having less impact on cardiac autonomic function, PFA was associated with superior arrhythmia-free survival compared to CBA in the SINGLESHOT CHAMPION trial.^11^ This finding suggests that modulation of the cardiac ANS may not be essential for procedural success after PVI. However, within the CBA group, patients who exhibited stronger ANS modulation, in particular greater early HRV suppression, had significantly fewer arrhythmia recurrences. This observation aligns with findings from the CIRCA-DOSE study, where greater early reduction of HRV post-ablation was similarly associated with improved rhythm outcomes.^7^ A possible explanation for this apparent paradox is that pronounced ANS modulation following CBA may reflect more extensive and transmural lesions that extend to the epicardial GP. In this context, cardiac ANS modulation may not be mechanistically required for success, but rather serve as a surrogate marker of lesion depth and durability, and thus of lower risk for PV reconnection.

This interpretation is further supported by findings from the AFACT study, which included 240 patients undergoing thoracoscopic AF surgery.^9^ Patients with paroxysmal AF received PVI alone, whereas those with persistent AF underwent a more extensive lesion set. All participants were randomized to additional epicardial ablation of major GP and Marshall’s ligament vs. standard ablation without autonomic targets. While GP ablation achieved complete suppression of evoked vagal responses, it did not improve arrhythmia-free survival at 1 year.

On the other hand, because PFA induces significantly less autonomic modulation, markers such as HRV suppression or heart rate changes cannot be reliably used as indicators of lesion quality or transmurality in this setting. Unlike thermal ablation, where ANS effects may serve as a surrogate for lesion extent, the absence of such changes with PFA reflects its neural tissue–sparing properties rather than insufficient lesion formation. PFA typically produces more antral and wider lesions, which—together with potentially greater lesion completeness—may contribute to more durable PVI, as observed in mandated remapping procedures and thereby contribute to the lower arrhythmia recurrence rates observed in the SINGLE SHOT CHAMPION trial.^20,21^ Nevertheless, adequate tissue contact during PFA remains essential to ensure durable, transmural myocardial lesions and achieve effective PVI, which may be assessed using intracardiac echocardiography.^22,23^

### Limitations

Several limitations should be acknowledged. First, ICMs were inserted only after the ablation procedure, precluding intra-individual assessment of acute autonomic changes. As such, our analysis is limited to between-group comparisons and time-dependent recovery to pre-ablation values cannot be asserted. However, given the randomized controlled design, the reported comparisons remain valid. Second, the findings apply specifically to the PFA system used in this study. Variability in pulsed field strength, waveform parameters, and catheter configuration across PFA technologies limits generalizability. Third, follow-up was limited to 12 months; longer-term data are required to assess the durability of both arrhythmia-free survival and autonomic effects. Fourth, while PFA achieves good outcomes despite minimal ANS modulation, it remains unclear whether the addition of GP ablation could further improve arrhythmia-free survival or offer specific benefits in select cohorts, such as patients with vagally mediated AF. Finally, this analysis represents a substudy of a larger trial, and the results should be interpreted within that context.

## Conclusions

In this randomized trial with continuous rhythm monitoring, PFA was associated with significantly less cardiac ANS modulation than CBA, yet achieved superior arrhythmia-free outcome. These findings challenge the notion that cardiac ANS modulation is a causal factor for the effect of PVI in patients with paroxysmal AF. Autonomic effects after PVI may serve as a surrogate marker of lesion quality in thermal ablation, but not in PFA.

## Supplementary Material

euag152_Supplementary_Data

## Data Availability

The data that support the findings of this study are available from the corresponding author upon reasonable request.
