# Supplementary material for: Autonomic effects and ablation success after pulsed field and cryoballoon ablation: a SINGLE SHOT CHAMPION substudy
Source: Europace. 2026 Jun 18;28(6):euag152. doi: 10.1093/europace/euag152 (PMC13318154; doi:10.1093/europace/euag152)
Supplement: euag152_Supplementary_Data [file euag152_supplementary_data.docx]

**Autonomic Effects of Pulsed Field Versus Cryoballoon Ablation: Insights from Continuous Rhythm Monitoring**

Laurent Roten, M.D.^1^*, Thomas Kueffer, Ph.D.^1^*, Patrick Badertscher, M.D.^2^, Peter Jüni, M.D.^3^, Sven Knecht, Ph.D.^2^, Gregor Thalmann, M.D.^1^, Nikola Kozhuharov, M.D.^1^, Philipp Krisai, M.D.^2^, Corinne Jufer, M.S.^1^, Jens Maurhofer, M.D.^1^, Dik Heg, Ph.D.^4^, Tiago V. Pereira, Ph.D.^3^, Felix Mahfoud, M.D.^2^, Ph.D., Helge Servatius, M.D.^1^, Hildegard Tanner, M.D.^1^, Michael Kühne, M.D.^2^, Christian Sticherling, M.D^2^, Tobias Reichlin, M.D.^1^

for the SINGLE SHOT CHAMPION Investigators

* These authors contributed equally to this work

^1^ Department of Cardiology, Inselspital, Bern University Hospital, University of Bern, Bern, Switzerland

^2^ Department of Cardiology, University Hospital Basel, University of Basel, Basel, Switzerland

^3^ Clinical Trial Service Unit and Epidemiological Studies Unit, Nuffield Department of Population Health, University of Oxford, Oxford, United Kingdom

^4^ Department of Clinical Research, University of Bern, Bern, Switzerland

Supplemental material:

Contents

[Supplemental Figure 1: Freedom from recurrence of atrial tachyarrhythmia during the period from 91 to 365 days. 2](#_Toc218606269)

[Supplemental Table 1: Medications at baseline, discharge, and follow-up 3](#_Toc218606270)

[Supplemental Table 2. Temporal analysis 4](#_Toc218606271)

[Supplemental Table 3. Differences in ablation characteristics 5](#_Toc218606272)

## Supplemental Figure 1: Freedom from recurrence of atrial tachyarrhythmia during the period from 91 to 365 days.


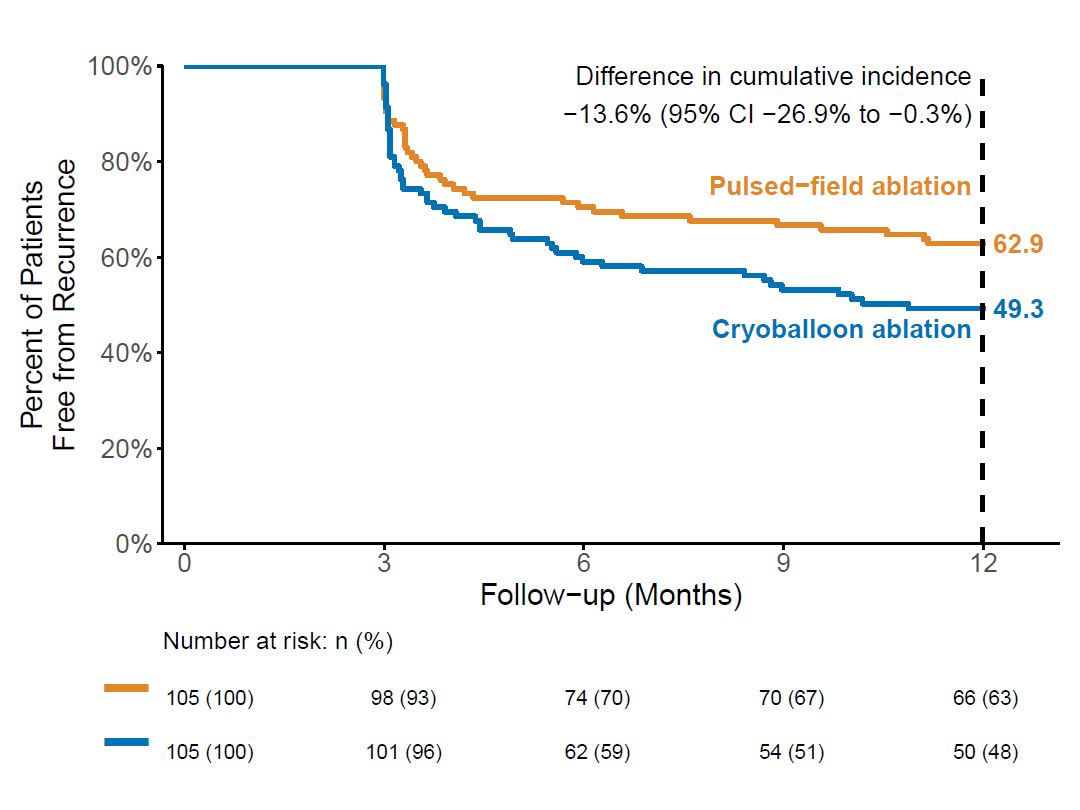


## Supplemental Table 1: Medications at baseline, discharge, and follow-up

| **Supplemental Table 1. Medications at baseline, discharge, and follow-up *** | | | |
| --- | --- | --- | --- |
|  | Overall | Pulsed field ablation | Cryoballoon ablation |
| N | 210 | 105 | 105 |
| **Medications at admission** |  |  |  |
| Antiarrhythmic drugs – no. (%) | 46 (21.9) | 22 (21.0) | 24 (22.9) |
| Amiodarone | 18 (8.6) | 8 (7.6) | 10 (9.5) |
| Class Ic antiarrhythmic drug | 23 (11.0) | 11 (10.5) | 12 (11.4) |
| Sotalol | 6 (2.9) | 4 (3.8) | 2 (1.9) |
| Beta-blocker | 133 (63.3) | 68 (64.8) | 65 (61.9) |
| Nondihydropyridine CCB | 13 (6.2) | 7 (6.7) | 6 (5.7) |
| Digoxin | 1 (0.5) | 1 (1.0) | 0 (0.0) |
| Anticoagulant use – no. (%) | 189 (90.0) | 93 (88.6) | 96 (91.4) |
| Direct oral anticoagulant | 183 (87.1) | 91 (86.7) | 92 (87.6) |
| Vitamin K antagonist | 6 (2.9) | 2 (1.9) | 4 (3.8) |
| **Medications at discharge** |  |  |  |
| Antiarrhythmic drugs – no. (%) | 25 (11.9) | 12 (11.4) | 13 (12.4) |
| Amiodarone | 8 (3.8) | 3 (2.9) | 5 (4.8) |
| Class Ic antiarrhythmic drug | 13 (6.2) | 7 (6.7) | 6 (5.7) |
| Sotalol | 4 (1.9) | 2 (1.9) | 2 (1.9) |
| Beta-blocker | 115 (54.8) | 58 (55.2) | 57 (54.3) |
| Nondihydropyridine CCB | 14 (6.7) | 7 (6.7) | 7 (6.7) |
| Digoxin | 0 | 0 | 0 |
| Anticoagulant use — no. (%) | 210 (100.0) | 105 (100.0) | 105 (100.0) |
| Direct oral anticoagulant | 205 (97.6) | 103 (98.1) | 102 (97.1) |
| Vitamin K antagonist | 5 (2.4) | 2 (1.9) | 3 (2.9) |
| **Medications at 3 months follow-up** |  |  |  |
| Antiarrhythmic drug — no. (%) | 14 (6.7) | 5 (4.8) | 9 (8.6) |
| Amiodarone | 7 (3.3) | 2 (1.9) | 5 (4.8) |
| Class Ic antiarrhythmic drug | 8 (3.8) | 3 (2.9) | 5 (4.8) |
| Sotalol | 0 | 0 | 0 |
| Beta-blocker | 109 (51.9) | 52 (49.5) | 57 (54.3) |
| Nondihydropyridine CCB | 19 (9.0) | 10 (9.5) | 9 (8.6) |
| Digoxin | 0 | 0 | 0 |
| Anticoagulant use — no. (%) | 183 (87.1) | 88 (83.8) | 95 (90.5) |
| Direct oral anticoagulant | 178 (84.8) | 86 (81.9) | 92 (87.6) |
| Vitamin K antagonist | 5 (2.4) | 2 (1.9) | 3 (2.9) |
| * Numbers are no. (%) or mean (SD) as appropriate. CCB denotes calcium channel blocker, and TIA transient ischemic attack. | | | |

## Supplemental Table 2. Temporal analysis

| **Supplemental Table2. Temporal analysis*** | | | | | | | | | |
| --- | --- | --- | --- | --- | --- | --- | --- | --- | --- |
| window | HRV | Difference in HRV | p-value | DHR | Difference in DHR | p-value | NHR | Difference in NHR | p-value |
| **PFA** | | | | | | | | | |
| Day 1-2 post ablation | 100 [80, 123] |  |  | 68 [63, 77] |  |  | 62 [56, 67] |  |  |
| 3rd month | 120 [99, 144] | 22 [5–37] | <0.001 | 73 [68, 80] | 5 [0–12] | <0.001 | 61 [57, 67] | 0 [-6–4] | 0.912 |
| 12th month | 116 [97, 143] | -1 [-12–6] | 0.105 | 74 [68, 81] | 0 [-3–2] | 0.516 | 61 [56, 66] | 1 [-1–3] | 0.049 |
| **CBA** | | | | | | | | | |
| Day 1-2 post ablation | 73 [59, 93] |  |  | 77 [68, 84] |  |  | 70 [63, 76] |  |  |
| 3rd month | 94 [82, 120] | 21 [7–42] | <0.001 | 78 [70, 87] | 3 [-3–8] | 0.005 | 67 [61, 72] | -2 [-6–2] | 0.001 |
| 12th month | 101 [85, 122] | 5 [-3–13] | <0.001 | 78 [71, 85] | -1 [-3–2] | 0.26 | 66 [61, 72] | -1 [-4–2] | 0.323 |
| * Autonomic parameters during 12-month follow-up after PFA or CBA. Values are median [interquartile range]. Changes represent median within-patient differences between consecutive follow-up windows. P-values reflect paired Wilcoxon signed-rank tests versus the preceding time interval. | | | | | | | | | |

## Supplemental Table 3. Differences in ablation characteristics

| **Supplemental Table 3. Differences in ablation characteristics of CBA patients*** | | | |
| --- | --- | --- | --- |
| Variable | Low HRV | High HRV | p |
| N | 52 | 52 |  |
| **Recurrence**† | 19 (36.5) | 33 (63.5) | 0.010 |
| Heart rate variability |  |  |  |
| Day 1-2 post ablation | 59 [47, 65] | 93 [82, 112] | <0.001 |
| 3rd month | 89 [76, 101] | 109 [91, 132] | <0.001 |
| 6th month | 89 [75, 101] | 117 [93, 137] | <0.001 |
| 12th month | 92 [79, 109] | 110 [96, 138] | <0.001 |
| **Ablation data**‡ |  |  |  |
| Number of freezes | 5.5 [5.0, 7.0] | 5.0 [5.0, 6.0] | 0.760 |
| LSPV | 1.0 [1.0, 2.0] | 2.0 [1.0, 2.0] | 0.590 |
| LIPV | 1.0 [1.0, 1.0] | 1.0 [1.0, 1.0] | 0.108 |
| RIPV | 1.0 [1.0, 2.0] | 1.0 [1.0, 2.0] | 0.870 |
| RSPV | 1.0 [1.0, 2.0] | 1.0 [1.0, 2.0] | 0.762 |
| Cumulative freeze duration – min | 16.0 [14.5, 21.0] | 16.0 [14.0, 19.5] | 0.766 |
| LSPV | 4.0 [3.0, 6.0] | 4.0 [3.0, 7.0] | 0.693 |
| LIPV | 3.8 [3.0, 4.0] | 4.0 [3.0, 4.0] | 0.250 |
| RIPV | 4.0 [3.0, 4.9] | 4.0 [3.0, 7.6] | 0.962 |
| RSPV | 3.0 [3.0, 4.0] | 4.0 [3.0, 4.0] | 0.751 |
| Nadir temperature – °C | -54.0 [-58.0, -52.0] | -53.0 [-56.0, -50.0] | 0.086 |
| LSPV | -50.0 [-53.0, -46.0] | -48.0 [-53.0, -45.0] | 0.760 |
| LIPV | -47.0 [-50.0, -44.0] | -45.0 [-48.0, -42.0] | 0.213 |
| RIPV | -49.0 [-52.0, -46.0] | -45.0 [-50.0, -42.0] | 0.012 |
| RSPV | -52.0 [-54.0, -49.0] | -50.0 [-53.0, -45.0] | 0.201 |
| * Numbers are no. (%) or median (IQR) as appropriate. CBA denotes Cryoballoon ablation, LIPV left inferior pulmonary vein, LSPV left superior pulmonary vein, RIPV right inferior pulmonary vein, and RSPV right superior pulmonary vein  † Defined as freedom from recurrent atrial tachyarrhythmia at 1 year using a blanking period of 90 days  ‡ Data for common ostia are reported under LSPV | | | |
